# Individualized Surgical Reconstruction of the Right Ventricle Outflow Tract in Double Outlet Right Ventricle With Mirror Image-Dextrocardia

**DOI:** 10.3389/fped.2021.611007

**Published:** 2021-02-19

**Authors:** Wangping Chen, Chukwuemeka Daniel Iroegbu, Xia Xie, Wenwu Zhou, Ming Wu, Xun Wu, Chengming Fan, Anton V. Borovjagin, Jinfu Yang

**Affiliations:** ^1^Department of Cardiovascular Surgery, The Second Xiangya Hospital of Central South University, Changsha, China; ^2^Department of Cardiovascular Surgery, The People's Hospital of Hunan Province, Changsha, China; ^3^Department of Biomedical Engineering, School of Medicine, University of Alabama at Birmingham, Birmingham, AL, United States

**Keywords:** double outlet right ventricle, dextrocardia, pulmonary stenosis, coronary artery, individualized reconstruction

## Abstract

**Introduction:** The purpose of this study was to report our experience in the surgical reconstruction of the right ventricular outflow tract in double outlet right ventricle with a major coronary artery crossing the right ventricular outflow tract in the presence of mirror image-dextrocardia.

**Methods:** From January 2005 to December 2019, 19 double outlet right ventricle patients (median age 4 years) with mirror image-dextrocardia and a major coronary artery crossing the right ventricular outflow tract received surgical repair. An autologous pericardial patch was used to enlarge the right ventricular outflow tract in four patients without pulmonary stenosis and three patients with mild pulmonary stenosis. A valved bovine jugular venous conduit was added to a hypoplastic native pathway in nine patients, among which six patients with moderate pulmonary stenosis received small-sized bovine jugular venous conduit implantation (diameter ≤ 16 mm). In comparison, a large-sized bovine jugular venous conduit (diameter >16 mm) was adopted in a total of three patients with severe pulmonary stenosis. Finally, three patients with preoperative pulmonary hypertension (mean pulmonary artery pressure ≥40 mmHg) did not undergo further intervention of right ventricular outflow tract due to the adequate outflow tract blood flow.

**Results:** There was no hospital mortality. One patient with sub-pulmonary ventricular septal defect and concomitant severe pulmonary hypertension died from respiratory failure 11 months after the operation. Kaplan-Meier survival was 94% at 5, 10 years. Within a mean echocardiographic follow-up of 6.9 ± 3.6 years, a total of two patients received reintervention due to valvular stenosis of the bovine jugular venous conduit (pressure gradient > 50 mmHg at 4 and 9 years) after surgical operation. Actuarial freedom from reoperation was 90 and 72% at 5 and 10 years, respectively. During the last echocardiographic follow-up phase, all the survivors were in NYHA class I.

**Conclusions:** Double outlet right ventricle with mirror image-dextrocardia is a rare and complicated congenital cardiac malformation. Surgical reconstruction of the right ventricular outflow tract should be individualized based on the degree of pulmonary stenosis and the specific anatomical features of each patient. Reconstructing the pulmonary artery using the various sizes of valved bovine jugular venous conduit is a safe and effective surgical method.

## Introduction

Double outlet right ventricle (DORV) is a type of complex congenital heart disease with abnormal ventriculoarterial connection (1). In the year 1972, Lev and colleagues were the first to describe the concept of DORV (2). The classical definition of DORV is defined as a congenital heart disease with: (i) both great arteries arising either entirely or predominantly (≥90%) from morphological right ventricle; (ii) the only outlet of left ventricle is a ventricular septal defect (VSD); (iii) the absence of aortic-mitral fibrous continuity. However, the last item is not usually an essential requirement for the diagnosis of DORV (1). Ikemoto and colleagues also reported DORV case with intact ventricular septum (3). Besides the traditional definition, some scholars also recommended defining DORV based on a 50% rule, where pulmonary artery entirely originates from the right ventricle, with more than 50% of the aorta originating from the right ventricle (4, 5). Anomalous coronary arteries crossing the RVOT often interferes with the surgical correction of RVOT obstruction, which significantly increases the morbidity and mortality (6, 7). Also, the operational difficulties also increase when combined with cardiac malposition. Dextrocardia is a rare type of congenital cardiac malposition in which the apex of the heart points to the right side, and a significant part of the heart is located in the right chest (8). But the circumstance of some acquired chest diseases causing pushing or pulling of heart toward the right is not defined as dextrocardia (9). The incidence of dextrocardia is 4 people per 100,000 live births (10). Dextrocardia with situs inversus, also called mirror image-dextrocardia, refers to a condition which the major visceral organs and the heart are mirror images of the norm (11, 12). Therefore, it is incredibly challenging to arrive at a clinically correct diagnosis and perform an ideal operation due to the anatomical variabilities and complexities faced with this cardiac disorder (13, 14). Importantly, amidst its rarity, late diagnosis and inadequate technical know-how only significantly increase the morbidity and mortality rate of the pathology. Thus, the present study aims to report the surgical reconstruction of the pulmonary artery in DORV patients with a major coronary artery crossing the right ventricular outflow tract (RVOT) in the presence of a mirror image-dextrocardia.

## Materials and Methods

### Patients

**Figure 1:**
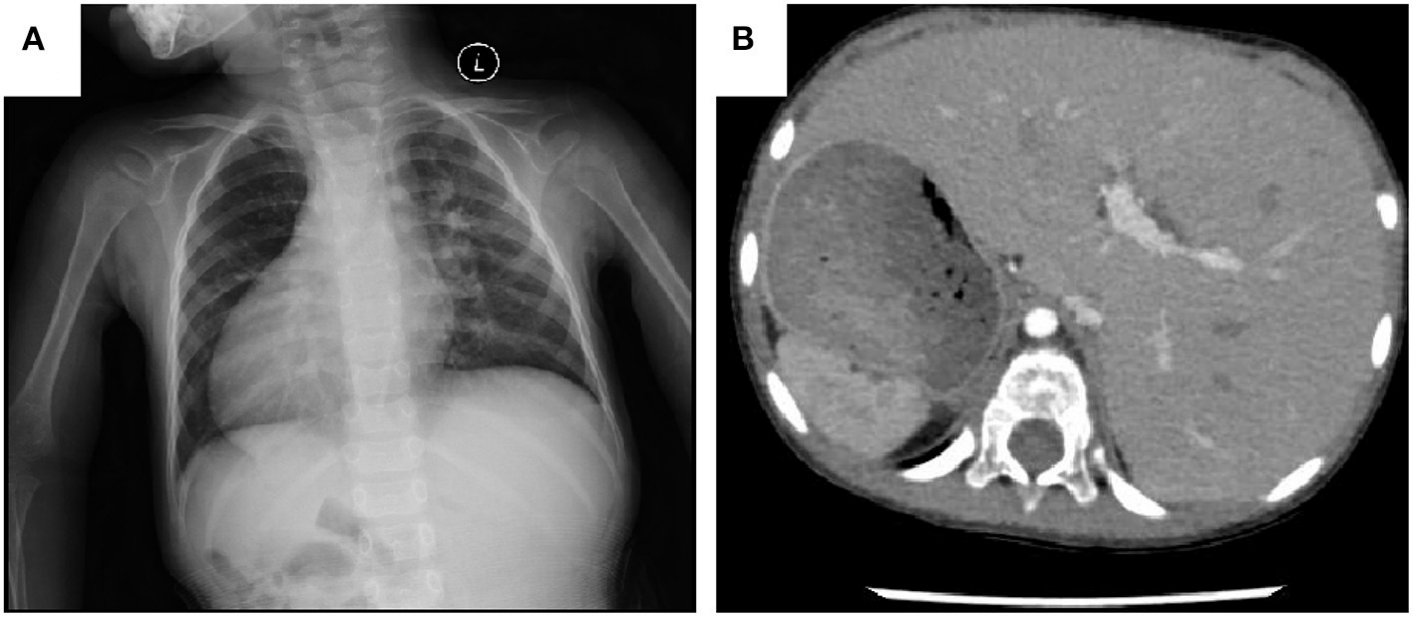
**(A)** Chest X-ray shows the cardiac apex directed to the right side. **(B)** CT scanning of the abdomen shows the liver on the left side, the stomach and spleen on the right.

**Figure 2:**
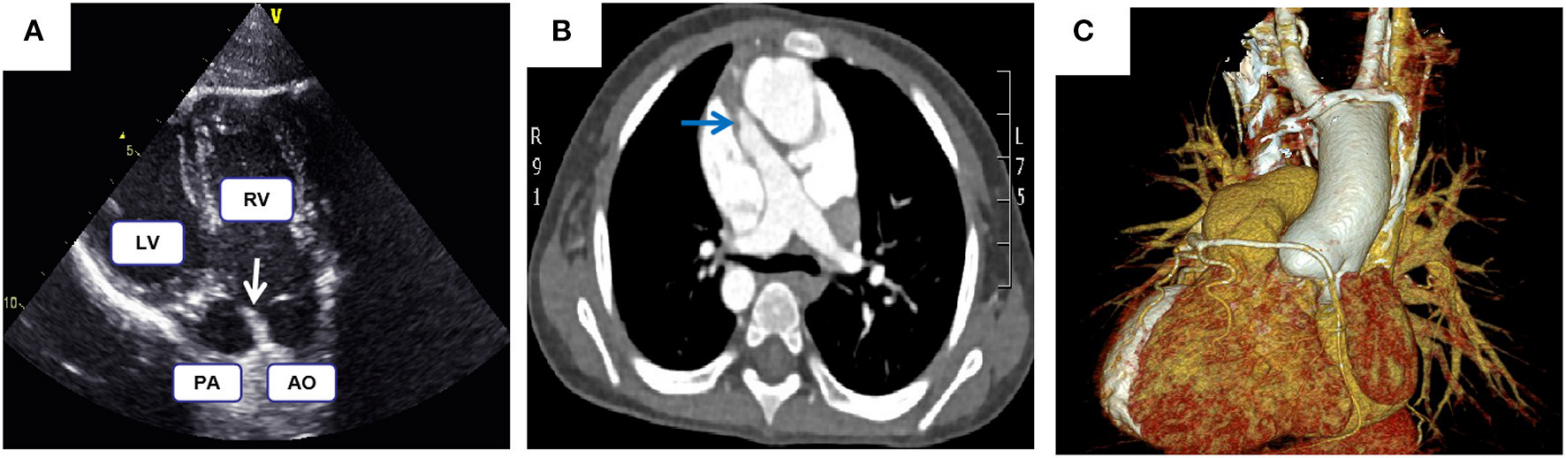
**(A)** Echocardiography demonstrates both the aorta and pulmonary artery originate from the right ventricle. **(B,C)** Computed tomography angiography shows malposed great vessels and anomalous right coronary artery looping around the pulmonary artery; Arrows highlight the pulmonary stenosis.

This retrospective study analyzed the clinical data of patients with DORV complicated by mirror image-dextrocardia and an anomalous coronary artery crossing the RVOT receiving surgical treatment in the Department of Cardiovascular Surgery, the Second Xiangya Hospital of Central South University, Changsha, China. Prior approval from the Research Ethics Committee of the Second Xiangya Hospital was obtained. Preoperative written and informed consent from all participants and their families was obtained. Methods used and applied to the research were carried out under relevant guidelines and regulations. From January 2005 to December 2019, a total of 19 patients underwent corrective surgery. There were 12 male and 7 female patients, with a median age of 4 years (range, 7 months to 15 years) and a median weight of 26 kg (range, 5.8–42 kg). All of the patients had a VSD, 12 cases were subaortic VSD (mainly under aorta), 3 cases were subpulmonary VSD (mainly under pulmonary artery), 3 cases were doubly committed VSD (approximate half under aorta) and 1 case was uncommitted VSD. Twelve patients had pulmonary stenosis, either valvular or subvalvular or both. Associated malformations were shown in Table 1. All the patients had an coronary artery crossing the RVOT. Different coronary artery variations included the left coronary artery arising anteriorly from the aorta and the anterior descending branch crossing the RVOT (*n* = 15), right coronary artery originating from left anterior descending branch and crossing the RVOT (*n* = 3), single right coronary artery sinus, major right coronary artery looping around pulmonary artery (*n* = 1). Cyanosis was observed in all patients. The typical images of dextrocardia and reversed visceral organs were demonstrated by chest X-ray and CT scanning of the abdomen, respectively (Figure 1). The diagnosis of DORV and accompanied cardiac malformations were verified by echocardiography, CT angiography, and surgical inspection. Figure 2 is representative auxiliary examination images of patients in the study.

**Table 1:** Associated cardiac malformations.

**Malformations**	**Patients**
Pulmonary stenosis	12
Patent ductus arteriosus	2
Atrial septal defect	3
Bilateral superior vena cava	2
Subaortic stenosis	1
PAPVC	1
Tricuspid valve regurgitation	2
Pulmonary hypertension	3

*PAPVC, Partial anomalous pulmonary venous connection*.

### Surgical Technique

All patients underwent operations through a median sternotomy on cardiopulmonary bypass with moderate hypothermia (28–30°C). The surgical inspection confirmed the findings of echocardiography and CT imaging. The apex of the heart pointed to the right side. The morphologic right atrium was found on the left while the morphologic left atrium was on the right. Situs inversus was verified by identifying both the superior vena cava and inferior vena cava on the left side, and by entering the morphologic right atrium. Two patients had bilateral superior vena cava. The lead surgeon stood on the left side of patients. Cannulation was performed by inserting the arterial cannula into the ascending aorta and the venous cannula into the inferior vena cava and left superior vena cava.

#### Intraventricular Tunnel Construction

In all patients, a right ventriculotomy incision was made. The orientation of ventriculotomy differed according to the exact path of coronary artery crossing the RVOT. A sizeable semi-arched patch cut from Gore-Tex vessel or Teflon or biological pericardial patch was used to direct blood shunted from the left ventricle through the VSD into the aorta. In three patients, the VSD was restrictive and was enlarged by incising the septum in its superior and lateral margins. For patients with sub-pulmonary and non-committed VSD, appropriate resection of the outlet septum was made when necessary to create an unobstructed and better-aligned intraventricular tunnel. Intraoperative transesophageal echocardiography was performed to ensure an unhindered left ventricle outflow tract.

#### RVOT Reconstruction

**Figure 3:**
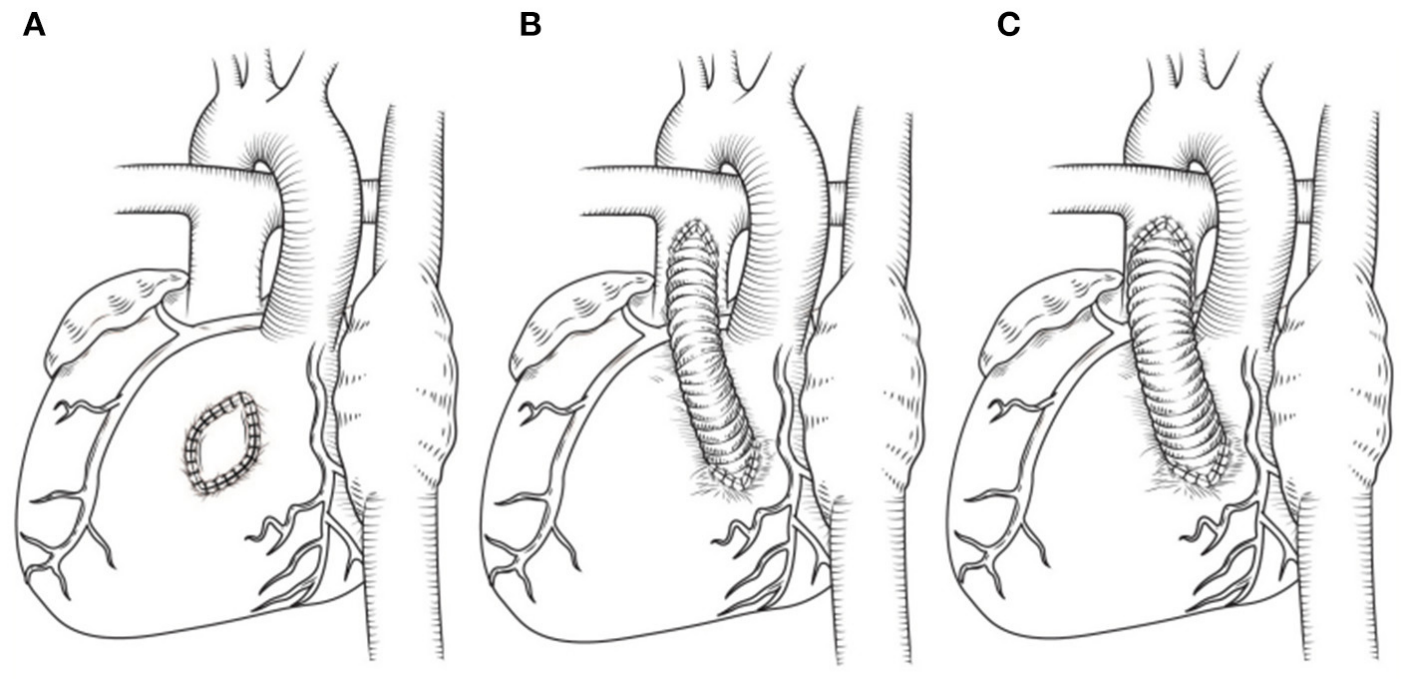
**(A)** Patch enlargement of RVOT. **(B,C)** Different size of BJVC adopted by patients with various degree of pulmonary stenosis and pulmonary anulus hypoplasia. For a given patient, more severe the pulmonary stenosis and smaller pulmonary artery anulus appeared, the larger size of BJVC was used.

The RVOT had to be enlarged by autologous pericardial patch due to the obstruction resulting from protrusion of the intraventricular tunnel in four patients without pulmonary stenosis (Figure 3A). For a given patient with pulmonary stenosis, the pulmonary artery reconstruction method was mainly determined by the degree of pulmonary stenosis, exact course of coronary artery crossing the RVOT and the size of pulmonary valve anulus. Resection of hypertrophied muscles, in addition of some kind of valvulotomy/plasty and the RVOT patch was adopted in a total of three patients with mild subvalvular and/or valvular pulmonary stenosis (Figure 3A). Besides, a transannular patch was not required with the patients, as mentioned above, as the pulmonary valve annulus was adequate. An valved BJVC was implanted between the right ventricle and the main pulmonary artery as a second pathway (apart from the innate pulmonary artery) in nine patients due to the proximity of the anomalous coronary artery to the hypoplastic pulmonary valve anulus and its inability to adequately relieve the marked subvalvular and valvular pulmonary stenosis (Figures 3B,C). We defined a mathematical formula as a reference to choice of proper size BJVC based on the fact that total blood flow volume of per cross-sectional area is equal as follows:

$$\begin{array}{l} {\left(\frac{R_{1}}{2}\right)}^{2}\pi +{\left(\frac{R_{2}}{2}\right)}^{2}\pi ={\left(\frac{R_{0}}{2}\right)}^{2}\pi \end{array}$$

where R1, R2, R0 represent the diameter of patient's innate pulmonary artery anulus, BJVC and normal pulmonary artery anulus, respectively. The diameter of normal pulmonary artery anulus is usually determined according to the body length and body surface area of patients (15). Based on this formula, we could calculate the size of BJVC as:

$$\begin{array}{l} R_{2}=\sqrt{R_{0}^{2}-R_{1}^{2}} \end{array}$$

Therefore, for a given patient, more severe the pulmonary stenosis and smaller pulmonary artery anulus appeared, the larger size of BJVC was used. With the case series in our study, a small-size valved BJVC (diameter ≤ 16 mm) was implanted in a total of six patients with moderate pulmonary stenosis, while a large-sized valved BJVC (diameter > 16 mm) was implanted in a total of three patients with severe pulmonary stenosis. Concomitant procedures were shown in Table 2. All of the patients who underwent conduit implantation received anticoagulation therapy with low-dose aspirin for 6 months post-surgery.

**Table 2:** Concomitant procedures.

**Procedure**	**Patients**
PDA ligation or suture	2
ASD closure	3
Subaortic resections	1
Tricuspid valvuloplasty	2
Right superior vena cava ligation	2
PAPVC repair	1
VSD enlargement	3
Pacemaker implantation	1

*PDA, Patent ductus arteriosus; ASD, Atrial septal defect; VSD, Ventricle septal defect; PAPVC, Partial anomalous pulmonary venous connection*.

### Statistical Analysis

Data are expressed as the mean ± SE and median. All statistical calculations are performed using the Statistical Product and Service Solutions 20 software (SPSS Institute). The Kaplan-Meier curve was used for the analysis of survival and freedom from reoperation for RVOT obstruction. The critical alpha level was set at *p* < 0.05.

### Ethics Statement

Prior approval from the Research Ethics Committee of the Second Xiangya Hospital was obtained. Preoperative written and informed consent from all participants and their families was obtained.

## Results

### Operative Details

There were no hospital deaths. A permanent artificial cardiac pacemaker was implanted in a patient due to a complete atrioventricular block after the operation. Exploratory thoracotomy was carried out in one patient due to excessive bleeding (25 ml/kg/h), which was from the anastomotic junction between the main pulmonary artery and the BJVC. The recovery period for all other patients was uneventful. Postoperative examination showed a complete and satisfactory repair of the cardiac malformations. The operative detail data were showed in Table 3.

**Table 3:** Postoperative data.

	**Value**
Cardiopulmonary bypass time (min)	115 ± 42
Aortic arrest time (min)	78 ± 35
Hospital residence time (d)	18 ± 3
ICU residence time (d)	5.2 ± 2.8
Mechanical ventilatory time (h)	74 ± 16
Size of BJVC (mm)	14.5 ± 2.1

### Follow-Up and Outcomes

**Figure 4:**
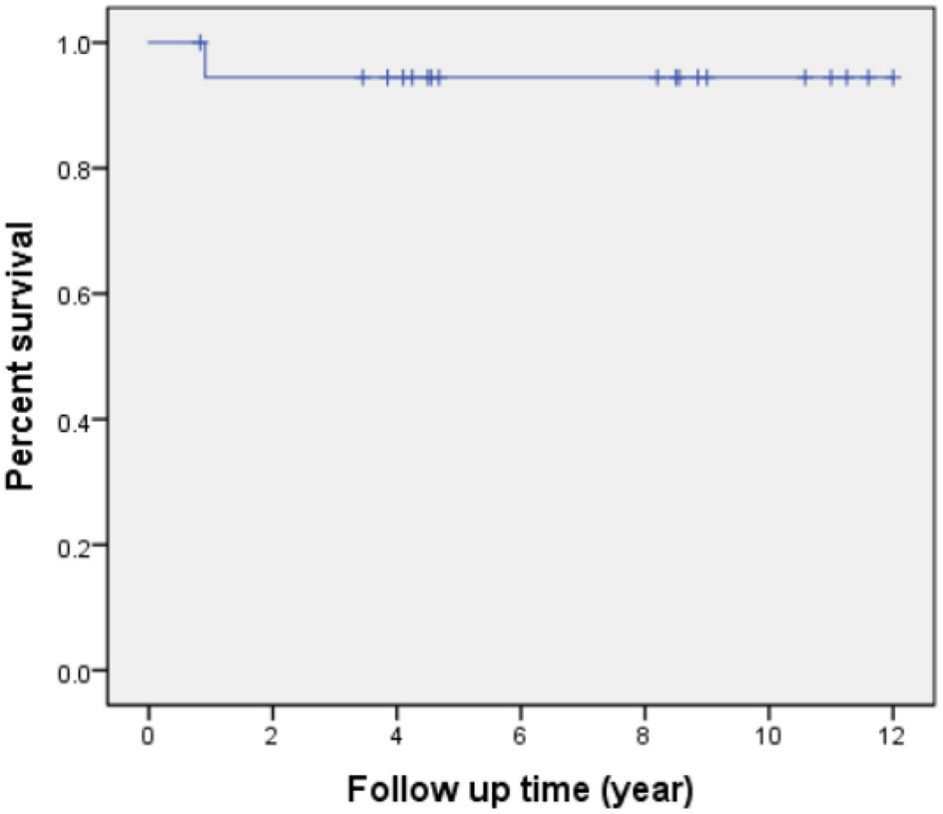
Patients survival following the operation.

**Figure 5:**
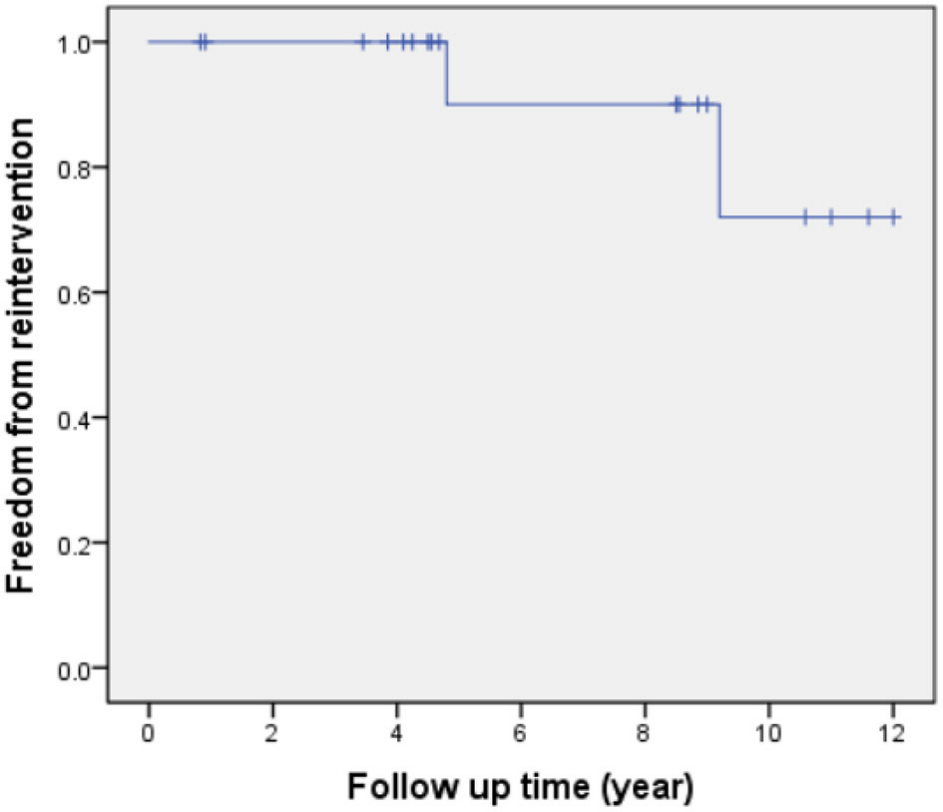
Freedom from reintervention of RVOT obstruction.

All patients received follow-up. The patient's cardiac functional condition was graded according to the New York Heart Association class. Physical examination and echocardiographic findings of patients were recorded. The indication of reintervention for RVOT included a right ventricle to pulmonary artery peak pressure gradient higher than 50 mmHg. The mean follow-up period after operation is 6.9 ± 3.6 years (range, 10 months to 12 years). There was, however, one patient with sub-pulmonary VSD and concomitant severe pulmonary hypertension who died from respiratory failure 11 months after the operation. Kaplan-Meier survival was 94% at 5, 10 years (Figure 4). Left ventricle function was normal in most patients throughout follow-up. According to the latest follow-up echocardiographic data, the left ventricular ejection fraction was 0.65 ± 0.04 (range, 0.62–0.70). Left ventricular outflow tract obstruction was not found in any patients during follow-up. However, a total of two patients had varying degrees of valvular stenosis of the BJVC. Between them, one patient presented with a pressure gradient of 55 mmHg and underwent balloon dilation due to weak valve activity, the other one underwent conduit replacement because of an 80 mmHg pressure gradient. Freedom from reintervention for RVOT obstruction was 90 and 72% at 5 and 10 years, respectively (Figure 5). At the latest follow-up, all surviving patients were in New York Heart Association class I and could engage in normal daily activities.

## Discussion

DORV is a complex cardiac malformation with variable pathological and hemodynamic features. Usually, a definitive preoperative diagnosis of DORV can be established by echocardiography and computed tomography angiography. With more intricate variations, however, the diagnostic gold standard can only be the surgical inspection after ventriculotomy. Depending on the location of VSD, the presence of pulmonary stenosis, and the degree of malposition of the great vessels, DORV mimics an unrestrictive VSD, a tetralogy of Fallot (TOF) or a transposition of the great arteries (TGA) clinically. Hence, the varying non-homogeneity results in an unending debate on the “proper” anatomical definition, classification, and the “right” surgical techniques to be used for DORV cases. In the present study, we reported a group of rare variations of DORV with mirror image-dextrocardia and a major coronary artery crossing the RVOT. Importantly, the surgical correction of the malformation was successfully performed in all cases. Through a right ventriculotomy incision, a sizeable semi-arched patch cut from Gore-Tex vessel or Teflon or biological pericardial patch was used to construct the intraventricular tunnel between the left ventricle and the aorta. The patch served as a baffle which directing blood shunted from the left ventricle through the VSD into the aorta. Based on the presence of pulmonary stenosis or not, we further divided the patients into two groups. A total of 12 patients showed pulmonary stenosis in our series, either valvular or subvalvular or both. For DORV patients combined with pulmonary stenosis, the relief of RVOT obstruction is a critical step for successful surgery. The presence of a major coronary artery crossing the obstructed outflow tract dramatically increased the morbidity and mortality during complete cardiac repair due to the possibility of injury to the coronary vessel (16, 17). In our study, the surgical strategy implemented (pulmonary artery reconstruction) was anatomically individualized for each patient. The location and course of coronary artery, the degree of pulmonary stenosis, and the size of pulmonary valve anulus are the most important factors that influence the surgical technique and outcomes. Where the pulmonary stenosis was mild, and the pulmonary valvular annulus was near echocardiographic standard size for age, the RVOT was relieved by sharply dissecting the hypertrophied muscle bundles via a ventriculotomy to avoid possible injuries to the coronary artery. An autologous pericardial patch was, however, also used to enlarge the RVOT. The aforementioned surgical strategy was carried out in a total of three patients. In a total of nine patients, however, the reconstruction of the pulmonary artery was accomplished using the valved BJVC to connect the ventricle and the pulmonary artery. The decision to use the valved BJVC to connect the ventricle and the pulmonary artery was due to proximity of the major coronary artery to the hypoplastic pulmonary valve annulus and its inability to relieve the RVOT obstruction adequately. Nonetheless, the innate pulmonary artery was preserved. To choose the proper size of BJVC, we defined a formula mathematically based on the fact that total blood flow volume of per cross-sectional area is equal as follows:

$$\begin{array}{l} {\left(\frac{R_{1}}{2}\right)}^{2}\pi +{\left(\frac{R_{2}}{2}\right)}^{2}\pi ={\left(\frac{R_{0}}{2}\right)}^{2}\pi \end{array}$$

where R1, R2, R0 represent the size of patient's innate pulmonary artery anulus, BJVC and normal pulmonary artery anulus, respectively. The size of normal pulmonary artery anulus is usually correlated to the body length and body surface area of patients (15). Based on this formula, the size of BJVC could be calculated as:

$$\begin{array}{l} R_{2}=\sqrt{R_{0}^{2}-R_{1}^{2}} \end{array}$$

Hence, for a given patient, more severe the pulmonary stenosis and smaller pulmonary artery anulus appeared, the larger size of BJVC was used. In this study, a small size valved BJVC (diameter ≤ 16 mm) was implanted in 6 patients with moderate pulmonary stenosis. In contrast, however, a larger size valved BJVC (diameter > 16 mm) was implanted in a total of 3 patients with severe pulmonary stenosis. Compared with the classic method of using BJVC to reconstruct the right ventricle and pulmonary artery connection (18–20), we preserved the innate pulmonary artery and added different size of BJVC as a second pathway according each patient's anatomical features. By doing so, we hoped that the innate pulmonary artery pathway would adequately stretch as the patient increases in age, thus rendering a future replacement of extracardiac conduit unnecessary. Crucial details were taken into consideration when the BJVC was implanted. Firstly, both the inflow and outflow end of the conduit were cut obliquely to form a hood over the rhombic incision in the pulmonary artery and right ventricle, which increased anastomotic area and decreased angulation. Importantly, the valve was implanted distally toward the pulmonary artery bifurcation with most of the outflow end of the conduit excised to avoid conduit valve distortion during sternal closure. Also, the length and path of the conduit should be appropriately considered to avoid sternal compression and overextension of the conduit. Firstly, the length of the conduit should be cautiously determined. An oversized conduit could distort transplantation, therefore, result in sternal compression. The conduit path should also be designed according to the morphological characteristics of the heart, and in most circumstances, it is placed at the right hand of the aorta. By doing so, after anastomosis, the central body part of the conduit would usually naturally extend to the right side in the shape of a “C,” partly avoiding the compression caused by chest closure. Besides, a Gore-Tex membrane could be sewn to the native pericardium before sternal closure to cover the conduit and other anterior cardiac structures, which will definitely reduce sternal compression and benefit re-entry when future surgery is needed. In our study, the overall mid-term outcome of the RVOT reconstruction was favorable, with a freedom from reintervention rate of 90 and 72% at 5 and 10 years, respectively.

Importantly, another surgical difficulty found with these patients was the presence of a mirror image-dextrocardia, which creates operational challenges such as the position of the surgeon, cannulation techniques, and the surgical approach to be adopted. Though rarely reported in the literature, dextrocardia represents <1% of all congenital heart defects (10), where the mortality rate remarkably increases as a result of late diagnosis especially with patients who are financially incapacitated (unfortunate case of one death in our study), inadequate technical know-how, and lack of access to adequate health-care facilities. The presence of dextrocardia results in a mirror image anatomy of the cardiac vasculatures as compared to the typical cardiac silhouette in healthy patients. Hence, detailed and rigorous preoperative anatomical assessment with adequate surgical planning is essential to prevent potential operative complications, thus decrease the overall mortality rate. As earlier mentioned, difficulties faced with venous cannulation for cardiopulmonary bypass due to interruption of the inferior vena cava significantly increases in dextrocardia patients (7). Thus, we carried out an enhanced preoperative CT to ascertain the likelihood of an interrupted inferior vena cava. However, there were no interrupted inferior vena cava with abnormal drainage in all cases. Hence, the classic cannulation technique was adopted by inserting the arterial cannula into the ascending aorta and the venous cannula into the vena cava, respectively.

## Conclusions

DORV with mirror image-dextrocardia and a major coronary artery crossing the RVOT is a rare and complicated congenital cardiac abnormality, which poses specific difficulties to the diagnosis and surgical corrections. The surgical reconstruction of the RVOT should be individualized based on the degree of the pulmonary stenosis and the particular anatomical features of each patient. Also, the use of different size valved BJVC in pulmonary artery reconstruction is safe and effective.

## Data Availability Statement

The raw data supporting the conclusions of this article will be made available by the authors, without undue reservation.

## Ethics Statement

The studies involving human participants were reviewed and approved by Research Ethics Committee of the Second Xiangya Hospital. Written informed consent to participate in this study was provided by the participants' legal guardian/next of kin. Written informed consent was obtained from the minor(s)' legal guardian/next of kin for the publication of any potentially identifiable images or data included in this article.

## Author Contributions

WC: conception and design of the work, acquisition, interpretation of data, and drafts the manuscript. CI and XX: acquisition and interpretation of data, and help with translation of manuscript. WZ: design of the work. MW, XW, and CF: acquisition and interpretation of echocardiography and CT data. CF: revised the manuscript. AB: drafts and translated the manuscript. JY: conception and design of the work, drafts the manuscript and made substantial revisions on it, and approved the final version. All authors contributed to the article and approved the submitted version.

## Conflict of Interest

The authors declare that the research was conducted in the absence of any commercial or financial relationships that could be construed as a potential conflict of interest.
